# A Case of Pancreatolithiasis Treated by a Combination of Extracorporeal Shock Wave Lithotripsy, Metallic Stent Placement, and Radiofrequency Ablation

**DOI:** 10.7759/cureus.94329

**Published:** 2025-10-11

**Authors:** Keisuke Kudo, Mitsuru Sugimoto, Yuki Kojima, Eriko Ikeda, Tetsuro Miwata, Atsushi Ishino, Jun Ushio, Hiromasa Ohira

**Affiliations:** 1 Gastroenterology, Fukushima Medical University, School of Medicine, Fukushima, JPN; 2 Gastroenterology, Hoshi General Hospital, Koriyama, JPN; 3 Gastroenterology, Jichi Medical University, Shimotsuke, JPN; 4 Digestive Disease Center, Showa Medical University Koto Toyosu Hospital, Toyosu, JPN

**Keywords:** chronic pancreatitis, extracorporeal shock wave lithotripsy, metallic stent, pancreatic duct stricture, pancreatolithiasis, radiofrequency ablation

## Abstract

For chronic pancreatitis (CP) with pancreatolithiasis, extracorporeal shock wave lithotripsy (ESWL), endoscopic stenting with plastic stents, and the endoscopic clearance of stone fragments are recommended treatment strategies. However, when a main pancreatic duct (MPD) stricture exists, pancreatolithiasis often becomes recurrent and refractory. However, the combination of radiofrequency ablation and metallic stent placement has recently been performed to treat malignant biliary stricture. In this case, we used a similar treatment in a patient with MPD stricture and recurrent pancreatolithiasis for the first time. A 46-year-old man visited a nearby doctor for abdominal pain. An MPD stone that was approximately 10 mm in length was observed in the pancreatic body on CT, and dilation of the distal MPD was observed. Pancreatolithiasis was found to be recurrent due to an MPD stricture at the pancreatic body following ESWL and endoscopic plastic stent placement. Therefore, we performed endoscopic stone clearance with RFA and endoscopic metallic stent placement. Three months later, the metallic stent was removed. After that, the patient was followed up, and there was no recurrence. The combination of RFA and metallic stent placement can serve as a novel treatment for symptomatic CP with MPD stricture and recurrent and refractory pancreatolithiasis.

## Introduction

Chronic pancreatitis (CP) is an inflammatory syndrome characterized by fibrosis, main pancreatic duct (MPD) stricture, and pancreatolithiasis. The etiology of CP mainly involves drinking alcohol and other factors (genetic mutation, autoimmune disorders, etc.) [1]. Pancreatolithiasis sometimes prevents the stream of pancreatic juice and can lead to abdominal pain, acute pancreatitis, and pancreatic pseudocysts [2,3]. Therapies such as endoscopic pancreatic stenting, endoscopic lithotripsy, and extracorporeal shock wave lithotripsy (ESWL) are indicated for the treatment of such cases of pancreatolithiasis [4]. On the other hand, pancreatic duct stricture often makes endoscopic lithotripsy difficult. In these difficult cases, ESWL is performed. However, MPD stones can recur because of MPD stricture [5,6]. Here, we report the first case in which recurrent MPD stones and pancreatolithiasis were treated by a combination of ESWL, endoscopic drainage with metallic stent placement, and endoscopic radiofrequency ablation (RFA).

## Case presentation

A 46-year-old man visited a nearby doctor for abdominal pain. He was diagnosed with acute pancreatitis and treated conservatively. An approximately 10 mm long MPD stone was observed in the pancreatic body, and dilation of the distal MPD was also observed on CT. The patient was referred to Hoshi General Hospital for medical care. He had a past history of alcoholic liver disease and had one liter of beer and one liter of liquor per day. After the above observations, he quit drinking. His gamma-glutamyl transpeptidase concentration was slightly elevated at 49 IU/L, but the other serum parameters were normal at the time of consultation.

**Figure 1:**
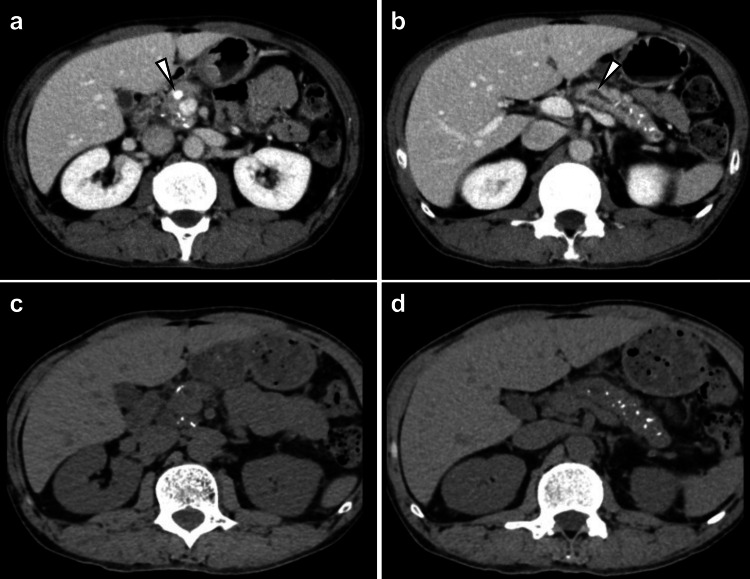
CT images before and after ESWL (a) Multiple calcifications were observed in the pancreatic head. The presence of a 10 mm long pancreatic ductal stone was confirmed in the main pancreatic head (white arrowhead). (b) Multiple calcifications were also observed in the pancreatic tail. The MPD was dilated on the distal side of the pancreatic stone (white arrowhead). (c) After ESWL, the pancreatic stone was destroyed. (d) Dilation of the distal pancreatic duct was relieved. CT: computed tomography, ESWL: extracorporeal shock wave lithotripsy, MPD: main pancreatic duct

Abdominal CT showed multiple calcifications on the pancreatic parenchyma, a 10 mm long MPD stone, and dilation of the distal MPD (Figure 1a-1b). According to the above results, his diagnosis was CP with pancreatolithiasis. Endoscopic lithotripsy was attempted; however, it was difficult due to an MPD stricture on the pancreatic body. Therefore, ESWL was performed, and the stone was destroyed (Figure 1c). Dilation of the MPD was also relieved (Figure 1d).

**Figure 2:**
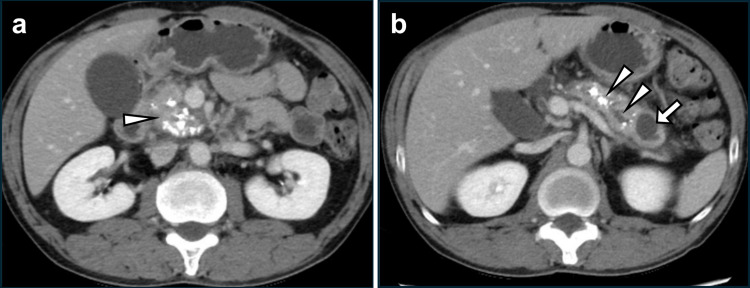
Imaging three months after the first treatment (a) Worsened pancreatic head calcification (white arrowhead). (b) Recurrence of pancreatolithiasis, a dilated distal MPD (white arrowheads), and a cyst on the pancreatic tail (white arrow) were also observed. MPD: main pancreatic duct

Three months after the first treatment, he experienced abdominal pain again. CT revealed that the calcification of the pancreatic head had worsened (Figure 2a). In addition, recurrence of an MPD stone in the pancreatic body, dilation of the distal MPD, and a 20 mm pancreatic tail cyst were observed (Figure 2b). A second endoscopic lithotripsy was attempted; however, it failed for the same reason (MPD stricture).

**Figure 3:**
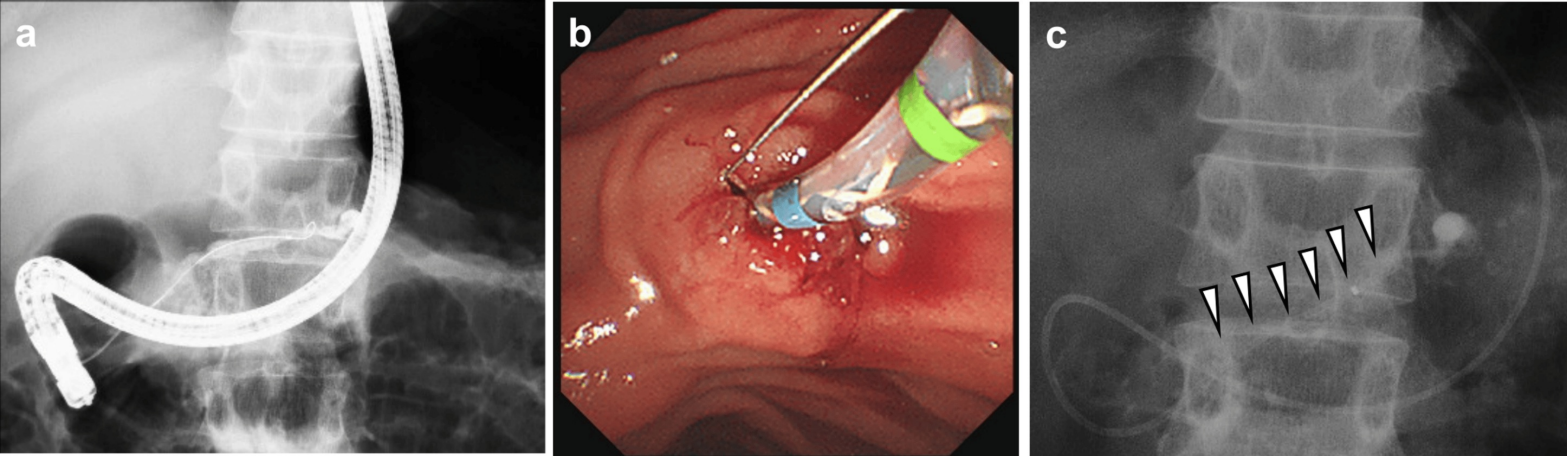
Endoscopic papillary treatment through the minor duodenal papilla (a) Pancreatic duct cannulation was performed from the minor duodenal papilla. (b) Incision of the minor pancreatic duct orifice. (c) ENPD for the prevention of impaction by MPD stone fragments (white arrowheads). ENPD: endoscopic nasopancreatic drainage

Therefore, after ESWL was performed four times, pancreatic duct cannulation was performed through the minor duodenal papilla (Figure 3a). After the minor pancreatic duct orifice was incised, an endoscopic nasopancreatic drainage (ENPD) tube was inserted to prevent acute pancreatitis resulting from the impaction of MPD stone fragments (Figure 3b-3c). After ESWL was performed two additional times, the crushed MPD stones were removed by endoscopic lithotripsy using a basket catheter through the minor duodenal papilla. Because the MPD stones were removed, endoscopic lithotripsy through the Vater papilla became possible. The major pancreatic duct orifice was incised, and endoscopic lithotripsy through the Vater papilla was performed. Endoscopic pancreatic stenting (7 Fr/7 cm) of both the MPD and the accessory pancreatic duct was performed.

**Figure 4:**
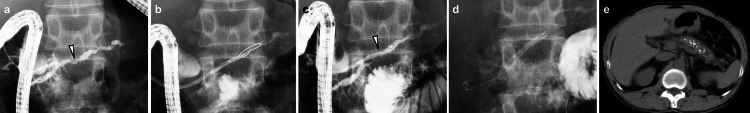
RFA and metallic stent placement (a) Residual MPD stricture on the pancreatic body (white arrowhead). (b) RFA of MPD stricture. (c) Relieved MPD stricture (white arrowhead). (d) Metallic stent placement to maintain MPD dilation. (e) Six months later, recurrence of pancreatolithiasis was not observed, and dilation of the distal MPD was relieved. RFA: radiofrequency ablation, MPD: main pancreatic duct

After endoscopic pancreatic stenting through both the major and minor duodenal papillae, ESWL was performed twice. On ERCP, MPD stricture was still observed on the pancreatic body (Figure 4a). To prevent recurrence of pancreatic ductal stones, RFA of the MPD stricture was performed (Figure 4b). After RFA, the MPD stricture was relieved (Figure 4c), and a 6 mm/6 cm fully covered metallic stent was placed (Figure 4d).

Three months later, the metallic stent was removed, and the outflow of contrast medium from the MPD was good. Six months later, recurrence of pancreatolithiasis and acute pancreatitis was not observed. Dilation of the distal MPD and pancreatic cyst was relieved (Figure 4e).

## Discussion

This report describes the first case in which ESWL, RFA, and metallic stent placement were used to alleviate pancreatic duct stricture and pancreatolithiasis. Though the first suggested treatment for CP is to stop drinking alcohol, recurrent MPD stones may not be effectively treated because of the established irreversible CP. Therefore, endoscopic treatment is necessary. The international conceptual model of CP includes four clinical stages: acute pancreatitis, recurrent acute pancreatitis, early CP, established CP, and end-stage CP [7]. Among them, established CP and end-stage CP are irreversible. According to the model, pancreatic calcification is associated with established CP. In the present case study, the patient had pancreatic calcification and was diagnosed with established CP, which is irreversible.

The American Society for Gastrointestinal Endoscopy (ASGE) guidelines for CP recommend ESWL for radiopaque stones >10 mm. The guidelines also recommend clearance of stone fragments <2-3 mm [4]. However, early recurrence of pancreatolithiasis can occur because of MPD stricture [6]. Recurrence also occurred in this case because of the MPD stricture. Therefore, treatment for MPD stricture was necessary.

The ASGE guidelines recommend endoscopic pancreatic stenting for patients with painful CP and MPD strictures [4]. For initial pancreatic drainage, the placement of the largest possible diameter plastic stent is recommended. In this case, we also used a plastic stent; however, the MPD stricture was not relieved. On the other hand, Lee et al. compared the use of plastic stents with that of fully covered metallic stents [8]. The rate of MPD stricture resolution was significantly higher in the metallic stent group than in the plastic stent group. Vila et al. summarized the outcomes of fully covered metallic stent placement for pancreatic ductal stenosis in a review article that examined twelve studies [5]. The mean duration of stent placement ranged from 2 to 7.5 months. The resolution rate of pancreatic duct stricture ranged from 67% to 100%. De novo stenosis was observed in 5.6% (12/214) of the patients. Therefore, metallic stents are potentially insufficient for some cases.

RFA is used mainly to treat malignant diseases; however, it has recently been applied to treat benign biliary and pancreatic strictures [9]. RFA might change and soften fibrous scar tissue; therefore, better therapeutic effects are expected for benign stricture [10]. In addition, when combined with metallic stent placement, RFA is expected to be more helpful in ameliorating pancreatic duct stricture and preventing the recurrence of pancreatolithiasis. In this case, repeated pancreatic duct stenting was performed for a benign MPD stricture and pancreatolithiasis; however, good progress was achieved by combining ESWL, RFA, and metallic stent placement.

The pancreatic pseudocyst was also relieved by transpapillary treatment, as described above. Endoscopic drainage is preferred over surgical drainage or percutaneous drainage because it is less invasive and is not associated with external tube infections. Endoscopic treatments for pancreatic pseudocysts include ERCP and endoscopic ultrasound (EUS)-guided cyst drainage (CD). With respect to EUS-CD, pseudocysts ideally adhere to the gastrointestinal wall to prevent leakage of cystic fluid [11,12]. Adhesion between the gastric wall and cyst was not observed in this case. Therefore, ERCP-guided treatment was selected and was determined to be effective.

## Conclusions

We report a case of CP, pancreatolithiasis, and MPD stricture that were cured by multimodal treatment with ESWL, endoscopic stone clearance, metallic stent placement, and RFA. Few reports have described the efficacy of RFA for benign pancreatic duct stricture. There are risky adverse events, such as pancreatitis and perforation of the pancreatic duct, and long-term outcomes are unknown. Adaptation and postoperative management strategies should be carefully selected. In the future, more studies are necessary to evaluate the efficacy of RFA for benign pancreatic duct stricture and pancreatolithiasis.
